# Oxalate of Potass in Puerperal Diseases

**Published:** 1855-11

**Authors:** 


Oxalate of Potass in Puerperal Diseases.—Dr. Ritter von Brenner strongly recommends this substance in inflammation of the peritoneum, uterus, or ovary, and especially in the metro-peritonitis of puerperal women. The formula is ℞ Aq. dist. ℥ vi., oxal. pot. vi., sacch. ʒij. M. A spoonful every hour.—(*Buchner's Report.* 1855. No. 4.) *Ib.*

